# Supplementary material for: Cryo- EM structure of the mycobacterial 70S ribosome in complex with ribosome hibernation promotion factor RafH
Source: Nat Commun. 2024 Jan 20;15:638. doi: 10.1038/s41467-024-44879-y (PMC10799931; doi:10.1038/s41467-024-44879-y)
Supplement: Supplementary file 1 — Supplementary Information [file 41467_2024_44879_MOESM1_ESM.pdf]

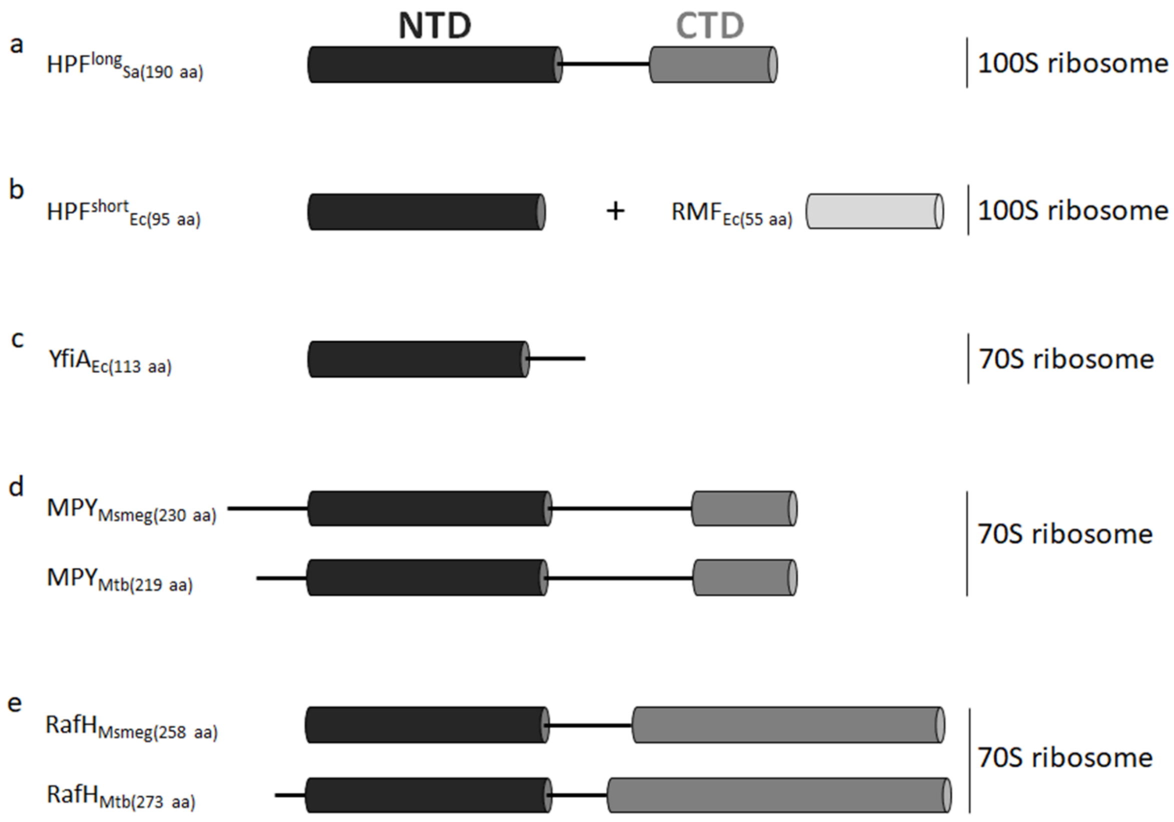

Supplementary Figure 1. Hibernation promotion factor domain organization. The domain organization of HPF, N-terminus domain (black), C- terminus domain (grey), connecting linker (black), and ribosome modulation factor (RMF) (grey) are shown. The right side shows the ribosome hibernation state 100S (disome) or 70S (monosome). (a) HPF^{long}, (b) HPF^{short} and RMF, (c) YfiA, (d) MPY from *M. smegmatis* and *M. tuberculosis*, (e) RafH from *M. smegmatis* and *M. tuberculosis* are shown.

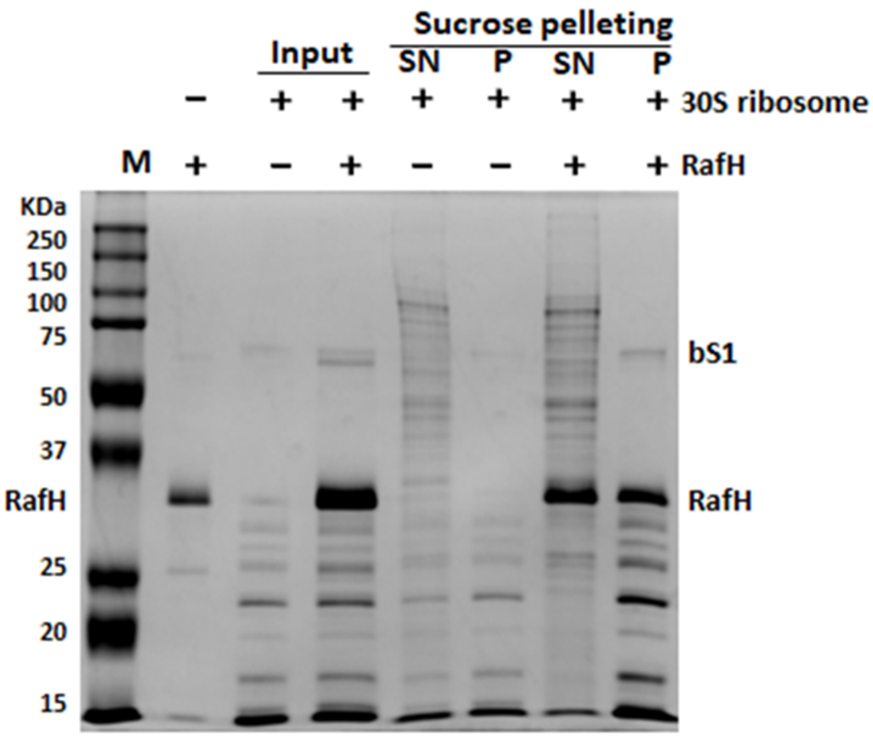

Supplementary Figure 2. Sucrose pelleting assay. The 30S ribosomes RafH complex formation by sucrose cushion pelleting assay analyzed on 12% SDS-PAGE, and stained with coomassie blue staining solution. Lane 1 - marker, lane 2 - pure RafH protein, lane 3, 4 - input, lane 5 to 8 - supernatant and pellet fraction after pelleting on a 0.8 M sucrose cushion. The source data for Supplementary Figure 2 is provided in the source data file.

a

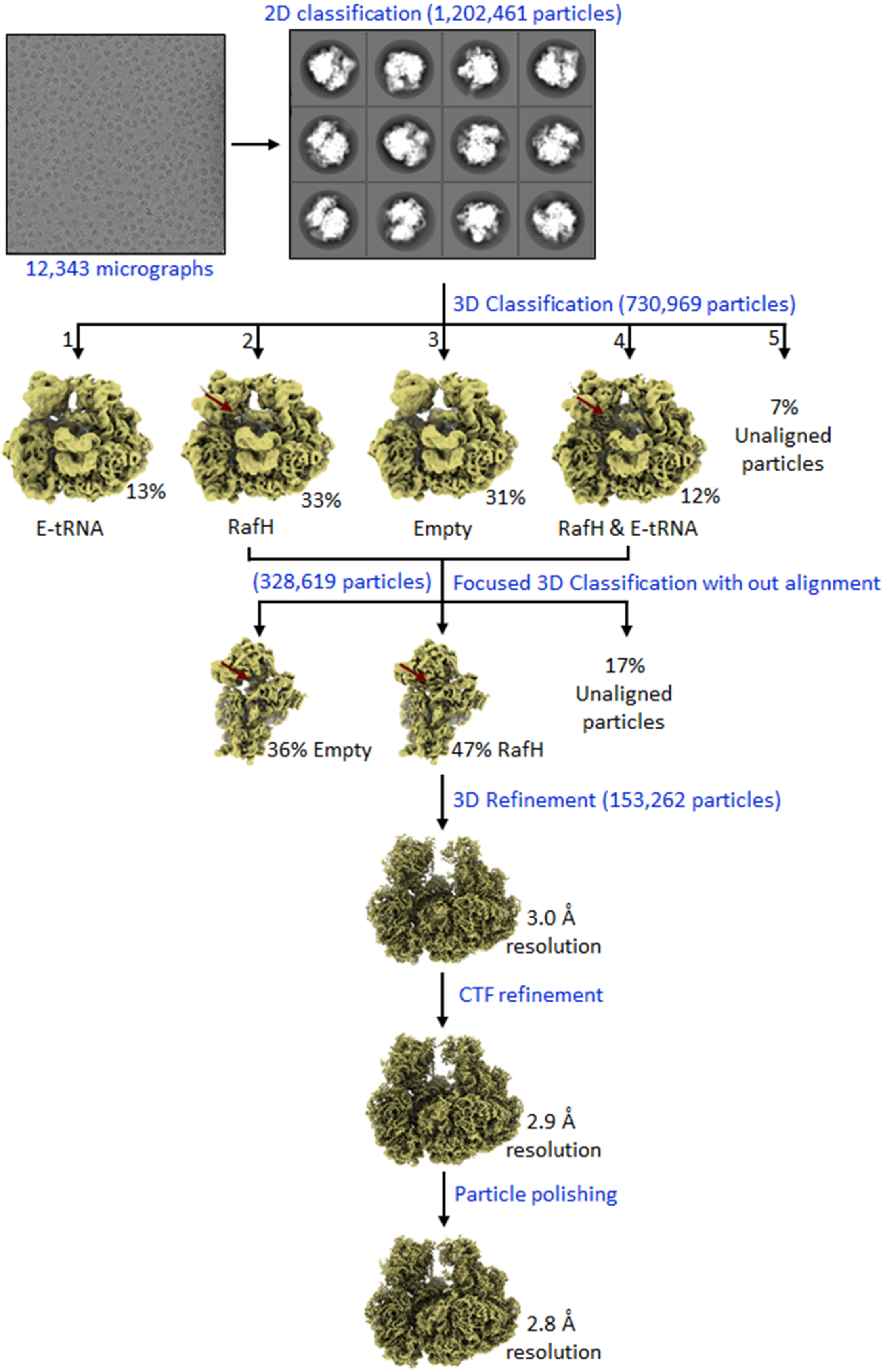
2D classification (1,202,461 particles)
12,343 micrographs
3D Classification (730,969 particles)
1
2
3
4
5
13%
E-tRNA
33%
RafH
31%
Empty
12%
RafH & E-tRNA
7%
Unaligned
particles
(328,619 particles)
Focused 3D Classification with out alignment
36% Empty
47% RafH
17%
Unaligned
particles
3D Refinement (153,262 particles)
3.0 Å
resolution
CTF refinement
2.9 Å
resolution
Particle polishing
2.8 Å
resolution

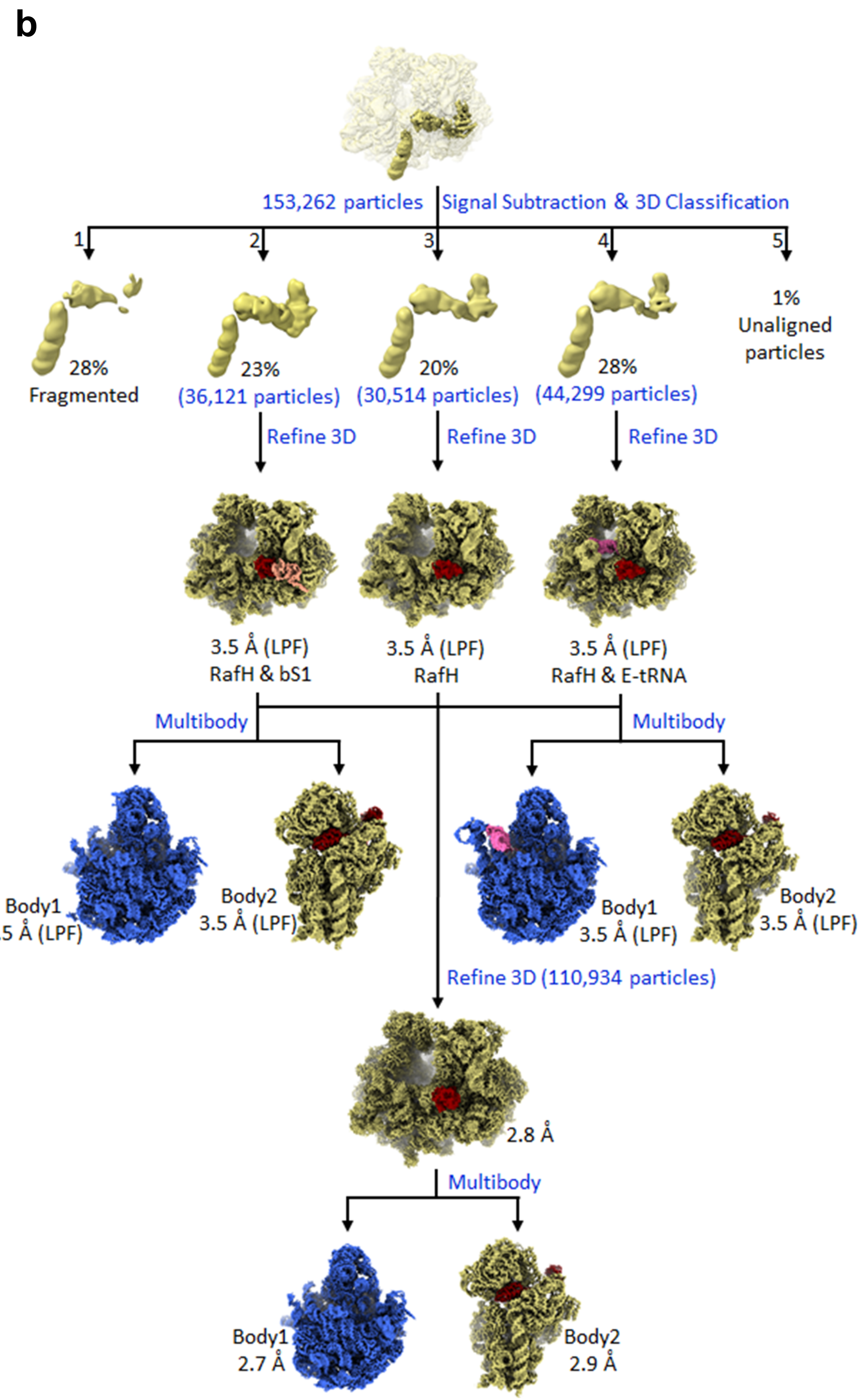

b
153,262 particles
Signal Subtraction & 3D Classification
1
2
3
4
5
28%
Fragmented
23%
(36,121 particles)
20%
(30,514 particles)
28%
(44,299 particles)
1%
Unaligned particles
Refine 3D
Refine 3D
Refine 3D
3.5 Å (LPF)
RafH & bS1
3.5 Å (LPF)
RafH
3.5 Å (LPF)
RafH & E-tRNA
Multibody
Multibody
Body1
3.5 Å (LPF)
Body2
3.5 Å (LPF)
Body1
3.5 Å (LPF)
Body2
3.5 Å (LPF)
Refine 3D (110,934 particles)
2.8 Å
Multibody
Body1
2.7 Å
Body2
2.9 Å

Supplementary Figure 3. Summary of single particle reconstruction. (a) 2D classification, 3D classification and initial consensus 3D maps and particle polishing are shown. The RafH NTD binding site is shown in a maroon arrow. (b) Signal subtraction with 3D classification without alignment and final multi-body refinement are shown. Maps were low pass filtered (LPF). All steps were performed using Relion 3.1.4.

Supplementary Table 1. Cryo-EM data collection, refinement, and validation statistics

	70S & RafH<br>EMDB-37551<br>PDB-8WHX	50S (body1)<br>EMDB-37552<br>PDB-8WHY	30 & RafH<br>(body 2)<br>EMDB-37565<br>PDB-8WIF	70S, RafH<br>& bS1<br>EMDB-37559<br>PDB-8WI7	50S (body1)<br>EMDB-37560<br>PDB-8WI8	30S, RafH &<br>bS1 (body2)<br>EMDB-37561<br>PDB-8WI9	70S, RafH<br>& tRNA<br>EMDB-37562<br>PDB 8WIB	50S & tRNA<br>(body1)<br>EMDB-37563<br>PDB-8WIC	30S, RafH<br>(body2)<br>EMDB-37564<br>PDB- 8WID
**Data collection & processing**									
Magnification	70,000								
Voltage (kV)	300								
Electron exposure (e–/Å^2)	1.34								
Defocus range (μm)	-0.5 – -3.0								
Pixel size (Å)	1.07								
Symmetry imposed	C1								
Initial particle images (no.)	1,202,461	1,202,461	1,202,461	1,202,461	1,202,461	1,202,461	1,202,461	1,202,461	1,202,461
Final particle images (no.)	110,934	110,934	110,934	36,121	36,121	36,121	44,299	44,299	44,299
Map resolution (Å)	2.8	2.7	2.9	3.5	3.5	3.5	3.5	3.5	3.5
FSC threshold	0.143	0.143	0.143	LPF	LPF	LPF	LPF	LPF	LPF
Map resolution range (Å)	2.5-5.0	2.5-5.0	2.5-5.0	3.5 -5.5*	3.5-5.5*	3.5-5.5*	3.5-5.5*	3.5-5.5*	3.5-5.5*
**Refinement**									
Initial model used (PDB code)	6DZI	8WHX	8WHX	8WHX	8WHX	8WHX	8WHX	8WHX	8WHX
Map sharpening *B* factor (Å^2)	-66.2	-55.98	-67.15	2.88	2.68	3.73	0.35	0.56	2.4
Model composition									
RNA nucleotides	4760	3237	1523	4760	3237	1523	4836	3313	1523
Protein residues	11094	6918	4176	11263	6918	4345	11094	6918	4176
R.m.s. deviations									
Bond lengths (Å)	0.01	0.01	0.01	0.01	0.01	0.01	0.01	0.01	0.01
Bond angles (°)	1.56	1.33	1.21	1.64	1.41	1.25	1.73	1.42	1.31
Validation									
MolProbity score	1.95	1.82	2.11	2.2	2.0	2.4	2.4	2.3	2.6
Clashscore	3.96	3.22	4.1	3.9	3.1	4.1	3.8	2.5	3.9
Poor rotamers (%)	2.1	2.98	2.5	2.5	2.3	2.7	2.6	2.4	2.8
Ramachandran plot									
Favored (%)	95.5	96.0	95.5	95.0	97.0	95.7	96.0	96.7	96.0
Allowed (%)	4.5	4.0	4.5	5.0	3.0	4.3	4.0	3.3	4.0
Disallowed (%)	0	0	0	0	0	0	0	0	0

* Ad-hoc Low pass filter (LPF) 3.5 Å was applied to the final map

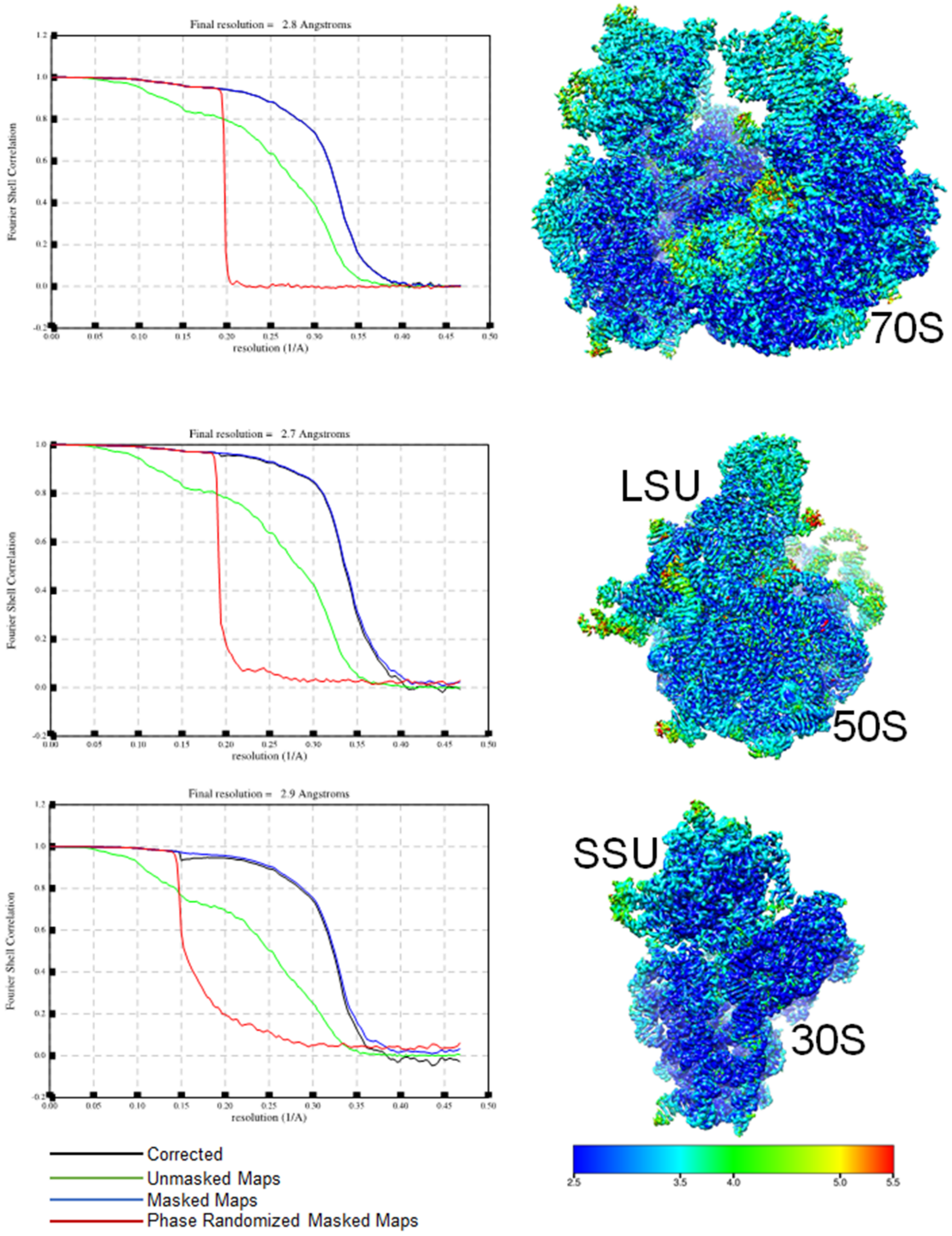

Supplementary Figure 4 Fourier Shell Correlation and local resolution. FSC for the 70S, 50S and 30S at top, middle, and bottom, respectively, shown in the left panel. The local resolution for the 70S, 50S, and 30S at the top, middle, and bottom, respectively, is shown in the right panel. The color bar for resolution is shown at the bottom right.

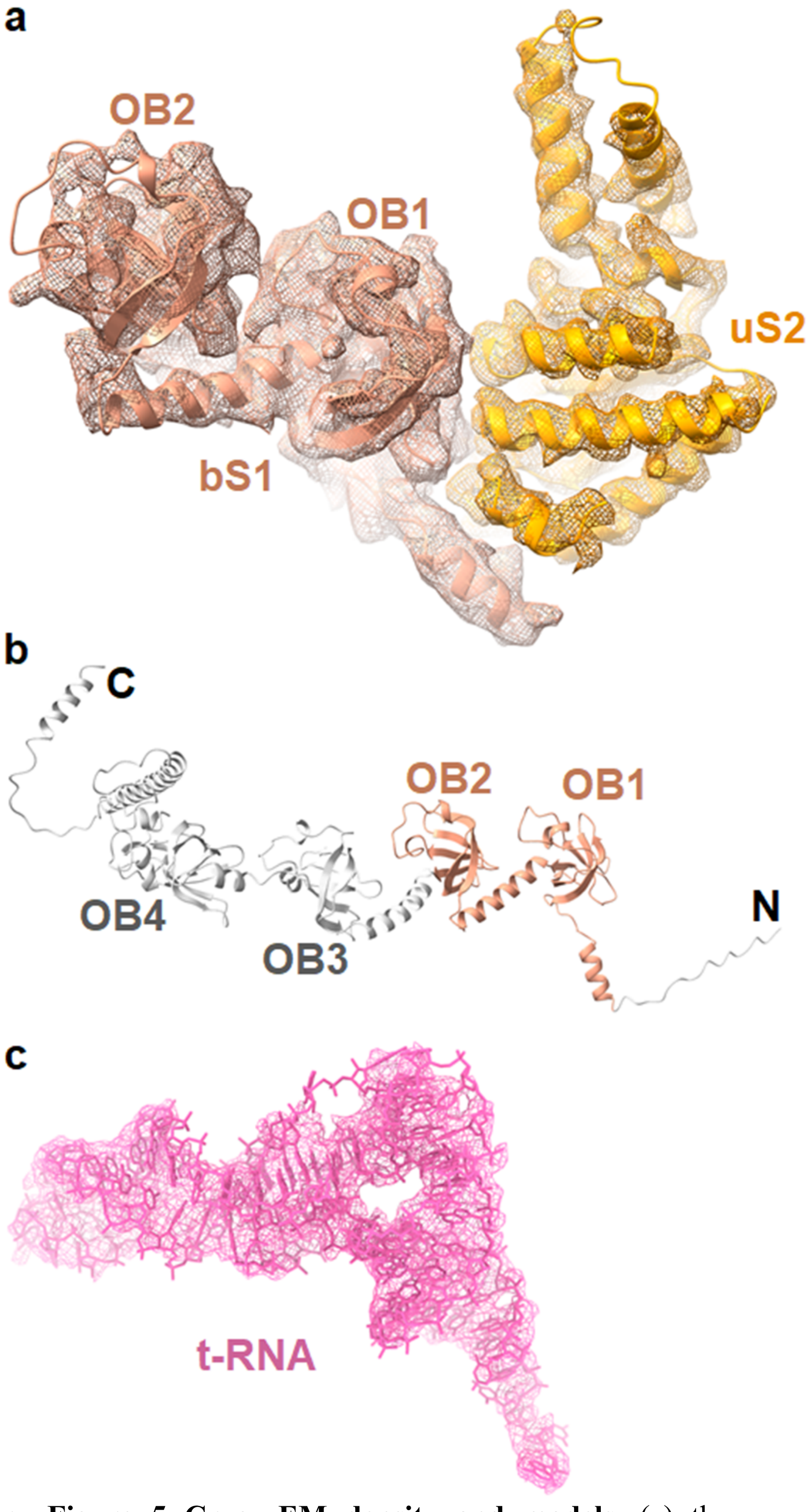

Supplementary Figure 5 Cryo- EM density and models. (a) the cryo- EM density corresponds to the OB1 and OB2 domains of bS1, and uS2 in mesh, and their models in the ribbon are shown. (b) the full-length structure of bS1 predicted using AlphaFold2. The domains the OB1 and OB2 (dark salmon) and unmodeled regions (grey) are shown. (c) the E- tRNA cryo- EM density in mesh (pink) and model in the sticks (pink) is shown.

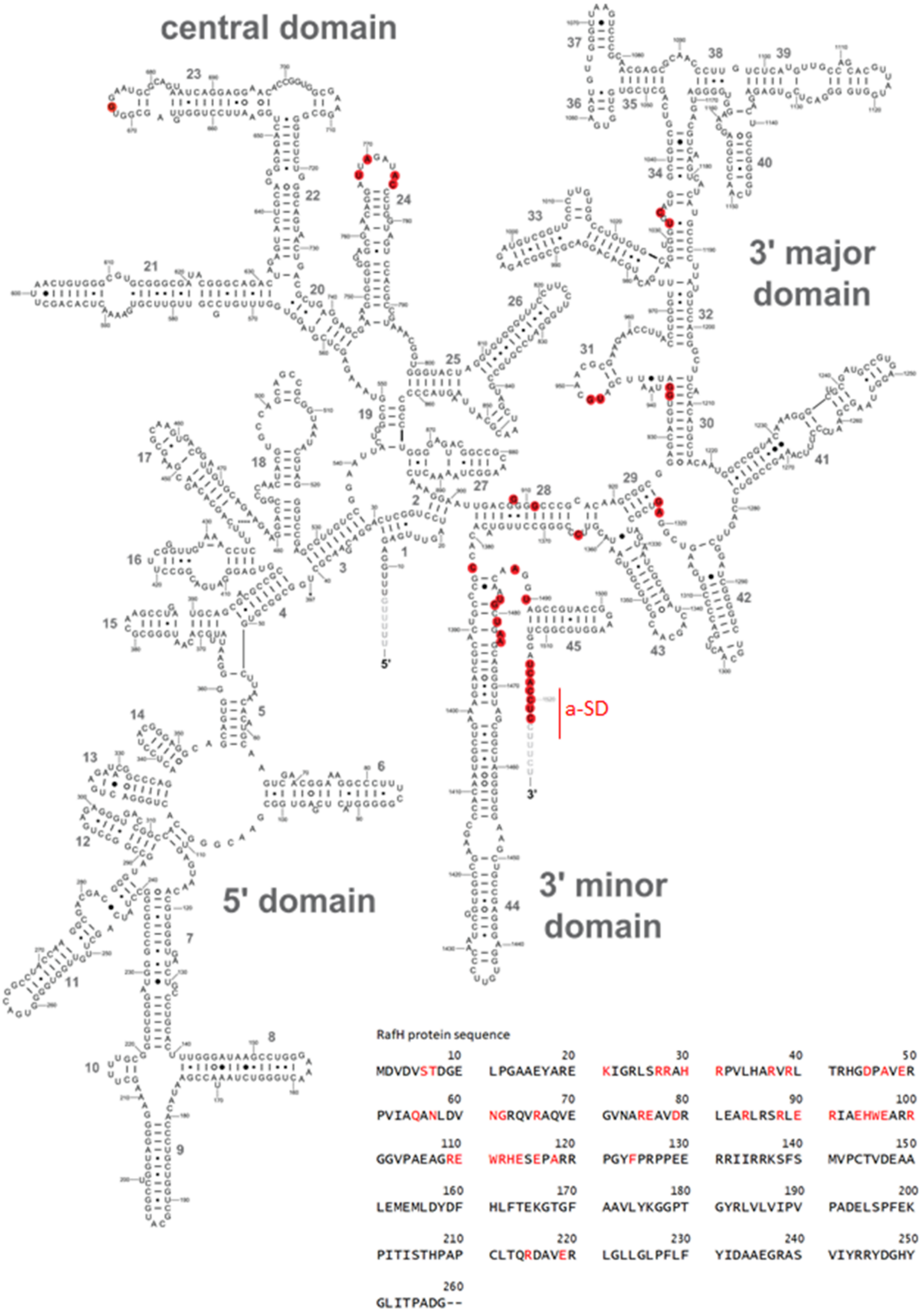

Supplementary Figure 6 16S rRNA and RafH interaction. The 16S rRNA 2D diagram and RafH sequence are shown. The RafH amino acid residues (red) and nucleotides (highlighted in red) are involved in interactions. The template for 16S rRNA 2D diagram was adopted from (Hentschel *et al.*, 2017)[40].

Supplementary Table 2 Interactions between RafH protein and rRNA & r-proteins and comparison with Mtb.

S.NO	Ms_RafH (Mt_RafH)	Ms_16S rRNA (Mt_16S rRNA)	Ms_r-proteins (Mt_r-proteins)
1	SER 6 (PHE 27)	G935 (G946)	
2	THR 7 (SER 28)	G936 (G947)	
3	LYS 21 (ALA 43)	G1478 (G1487)	
4	ARG 24 (ARG 46)	U1479 (U1488)	
5	ARG 27 (ASP 50)	A770 (A781)	
6	ARG 28 (ARG 51)	G1481/A770 (G1490/A781)	
7	HIS 30	A770 (A781)	
8	ARG 31	A35 E-tRNA	
9	LEU 34 (GLY 55)	A1321 (A1331)	
10	HIS 35 (GLY 55)	C1211 (C1222)	uS9 ARG 150 (uS9 ARG 150)
11	ARG 37 (ARG 58)	U947 (U958)	
12	ARG 39 (ARG 58)	U947 (U958)	
13	ASP 45	U1032/C1334 (U1043 /C1344)	
14	GLU 49	G510 (G521)	
15	GLN 55 (GLN 74)	G948 (G959)	
16	ASN 57 (ASN 76)	G948 (G959)	
17	ASP 59 (GLN 78)	-	uS9 ARG 150 (uS9 ARG 150)
18	ASN 61 (GLY 80)	A1321 (A1331)	
19	GLY 62 (ASP 81)	G1322 (G1332)	
20	GLN 64 (PRO 83)	-	uS9 LYS 149 (uS9 LYS 149)
21	ARG 66 (ARG 85)	G948 (G959)	
22	ARG 75 (ASP 94)	A1477 (A1486)	
23	GLU 76 (ASP 95)	A1477 (A1486)	
24	ASP 79 (ARG 97)	A1477 (A1486)	
25	ARG 80 (PRO 98)	A1476 (A1485)	
26	ARG 84 (ARG 102)	G1384 (G1394)	
27	ARG 88 (GLN 106)	C1383 (C1393)	
28	GLU 90 (VAL 108)	U1482 (U1491)	
29	ARG 91 (ARG 109)	C1382/G908 (C1392/G919)	
30	GLU 94 (ALA 112)	C34 E-tRNA	
31	HIS 95 (GLN 113)	C34 E-tRNA /U769 (U780)	
32	TRP 96 (TRP 114)	G673 (G685)	
33	GLU 97 (CYS 115)	C775/A774/U768 (C786/A785/U779)	
34	ARG 100	C775/U1490 (C786/U1499)	uS11 ARG 137, VAL 138 (uS11 ARG 137, VAL 138)
35	ARG 109 (ARG 117)	C1517 (C1526)	
36	GLU 110 (PRO 118)	U1516 (U1525)	
37	TRP 111 (TRP 119)	A1518	
38	ARG 112 (PRO 120)	G909/A1487 (G920/A1496)	

39	HIS 113 (ASP 121)	G911 (G922)	
40	GLU 114 (ARG 122)	C1365 (C1375)	
41	ARG 120 (ARG 125)	C1519/C1520	
42	PRO 121	U1521	
43	PHE 124	C1522	
43	ARG 215 (ALA 223)	-	bS18 GLU 44 (bS18 GLU 44)
44	GLU 219 (ASP 227)	-	bS18 ARG 45 (bS18 ARG 45)
**S.NO**	**Ms_RafH (Mt_RafH)**	**Ms_23S rRNA (Mt_23S rRNA)**	**Inter-subunit bridge**
1	ARG 75 (GLU 94)	A2137 (A2151)	B2a

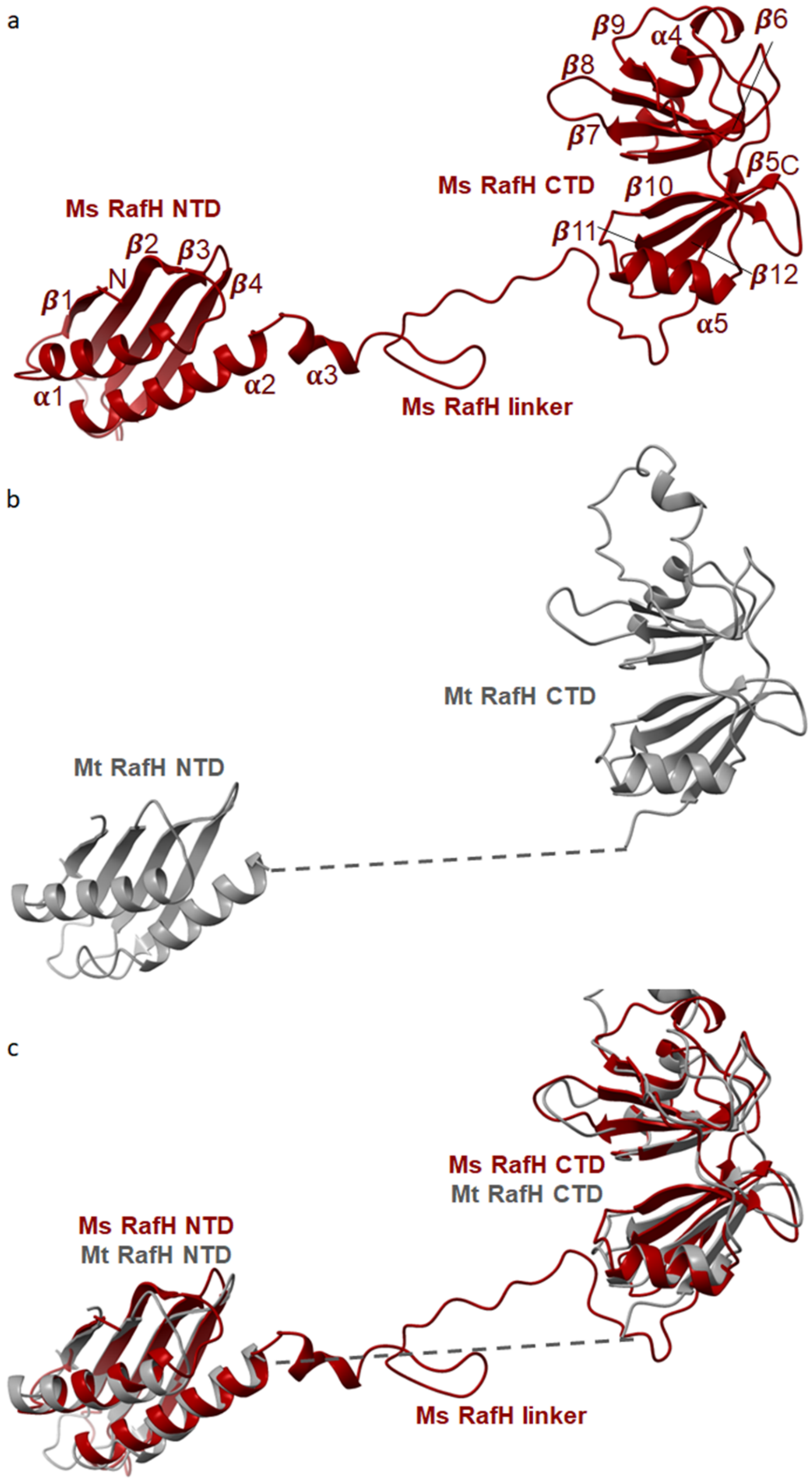

Supplementary Figure 7. *M. smegmatis* RafH full-length model and its structure comparison with *M. tuberculosis* RafH model. (a) the RafH full-length structure in ribbon style with labeled secondary structures, domains, and linker is shown. (b) the *M. tuberculosis* RafH N- and C- terminus domain fold predicted by AlphaFold2 and connecting linker shown in dotted line. (c) the N- and C- terminus domains of *M. smegmatis* RafH (red) superimposed with the N- and C- terminus domains of the *M. tuberculosis* RafH (grey).

```
Ms_RafH    1  .....................MDVDVSTDGELP.GAAEYAREKIGR.LS 26
Mt_RafH    1  MEPKRSRLVVCAPEPSHAREFPDVAVFSGGRANASQAERLARAVGRVLA 49
                                    ** * :.*.   . **   . :** *:

Ms_RafH   27 RRAHRPVLHARVRLTRHGDPAVER....PVIAQANLDVNGRQVRAQVEGV 72
Mt_RafH   50 DRG..VTGGARVRLTMAN......CADGPTLVQINLQVGDTPLRAQAATA 91
              *.   .  ******  .          *.:.* **:*..  :***.  .

Ms_RafH   73 NAREAVDRLEARLRSRLERIAEHWEARRGGVPAEAGREWRHESEPARRPG 122
Mt_RafH   92 GIDD.LRPALIRLDRQIVRASAQWC..........PRPWPDR..P.RR.. 125
             .  : :     **  :: *   :*            * *     * **

Ms_RafH  123 YFPRPPEERRIIRRKSFSMVPCTVDEAALEMEMLDYDFHLFTEKGTGFAA 172
Mt_RafH  126 RLTTPAE.ALVTRRKPVVLRRATPLQAIAAMDAMDYDVHLFTDAETGEDA 174
              .  *.*   : ***.. :  .*  :*   *: :***.****:  **  *

Ms_RafH  173 VLYKGGPTGYRLVLVIPVPADELSPF.........EKPITISTHPAPCLT 213
Mt_RafH  175 VVYRAGPSGLRLARQHHVFP..PGW.SRCRAPAGPPVPLIVNSRPTPVLT 221
             *:*:.**:* **.    * .   .             *: :.::*:* **

Ms_RafH  214 QRDAVERLGLLGLPFLFYIDAAEGRASVIYRRYDGHYGLITPADG..... 258
Mt_RafH  222 EAAAVDRAREHGLPFLFFTDQATGRGQLLYSRYDGNLGLITPT.GDGVAD 270
             :  **:*    ******: * * **..::* ****: *****: *

Ms_RafH      ...
Mt_RafH  271 GLA 273
```

Supplementary Figure 8. Sequence comparison between *M. smegmatis* and *M. tuberculosis* RafH. The alignment carried out using Clustal Omega, between *M. smegmatis* and *M. tuberculosis* RafH sequences. The * and . represents the identical and similar amino acid residues, respectively. The *M. smegmatis* amino acid residues interacting with 30S ribosomal subunit and corresponding residues in *M. tuberculosis* are boxed with a red background.

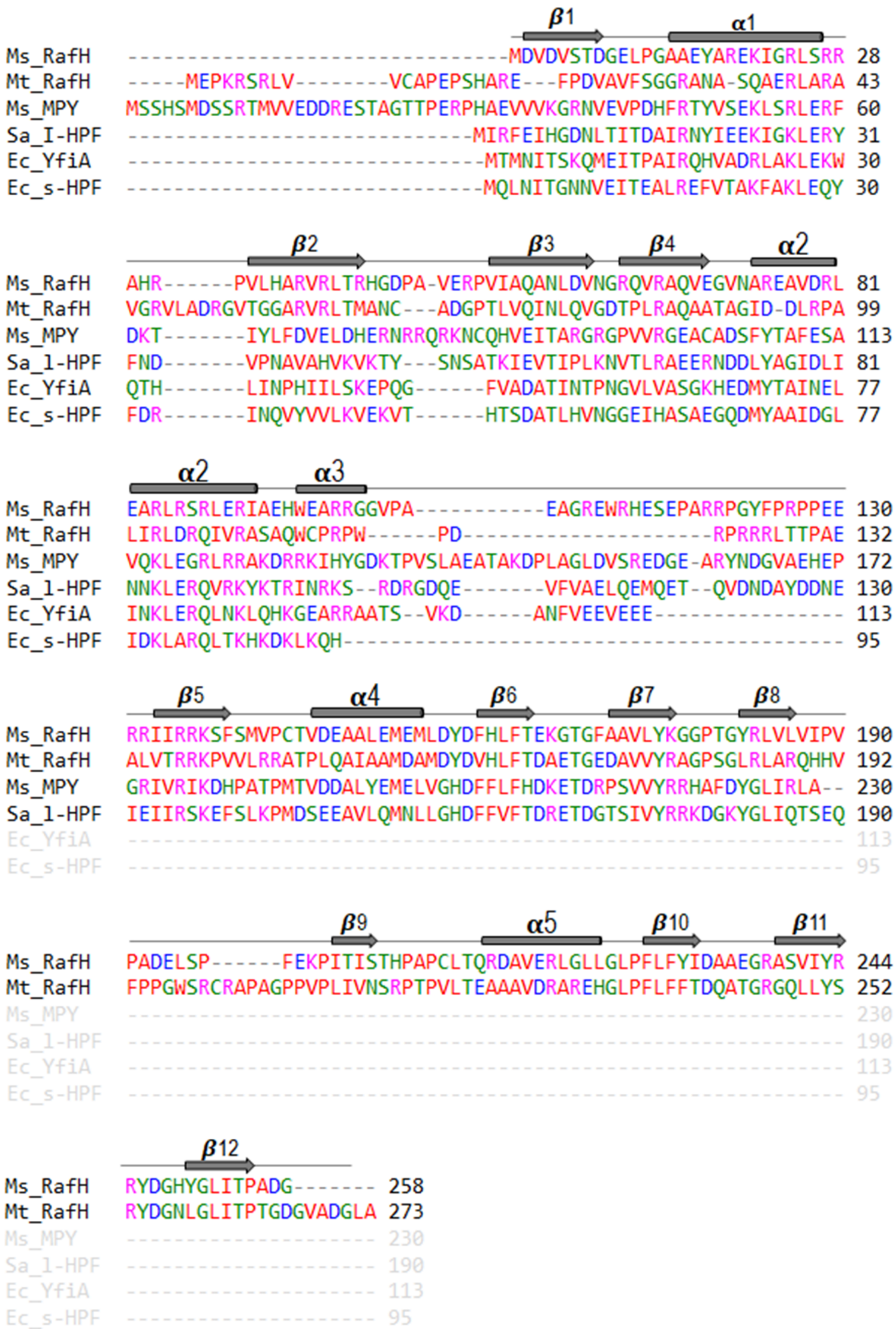

Supplementary Figure 9 Multiple sequence alignments. The multiple sequence alignment for *M. smegmatis* RafH (Ms_RafH), *M. tuberculosis* RafH (Mt_RafH), *M. smegmatis* MPY (Ms_MPY), *S. aureus* HPF^{long} (Sa_l-HPF), *E. coli* YfiA (Ec_YfiA) and *E. coli* HPF^{short} (Ec_s-HPF) with the secondary structures present in RafH (on top) is shown.

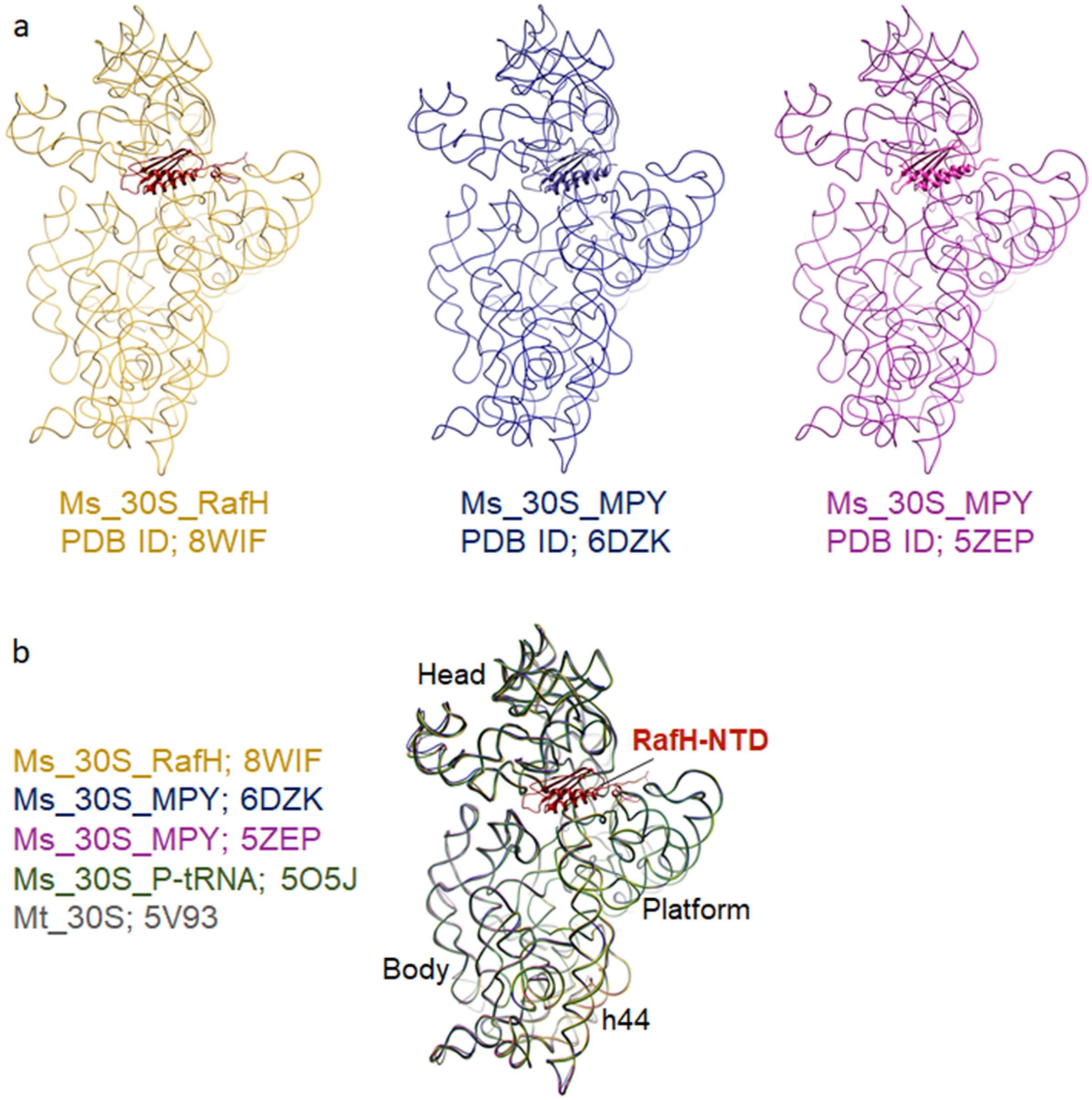

Supplementary Figure 10 Ribosomal small subunit comparison. (a) the structures of *M. smegmatis* SSU (Khaki) bound with RafH (red) (PDB ID; 8WIF) (left panel), bound with MPY (navy blue) (PDB ID; 6DZK), ribosome purified from Zinc starvation (middle panel), and bound with MPY (magenta) (PDB ID; 65ZEP), ribosome purified from stationary phase (right panel) are shown. For comparison, only RafH N- terminus domain is shown. (b) the structures shown in panel ‘a’ are superimposed with other structures of SSU from *M. smegmatis* bound with P- tRNA, translating ribosome, (green) (PDB ID; 5O5J) and SSU from *M. tuberculosis* (grey) (PDB ID; 5V93) are shown. For clarity, the MPY is not shown in superimposed structures.

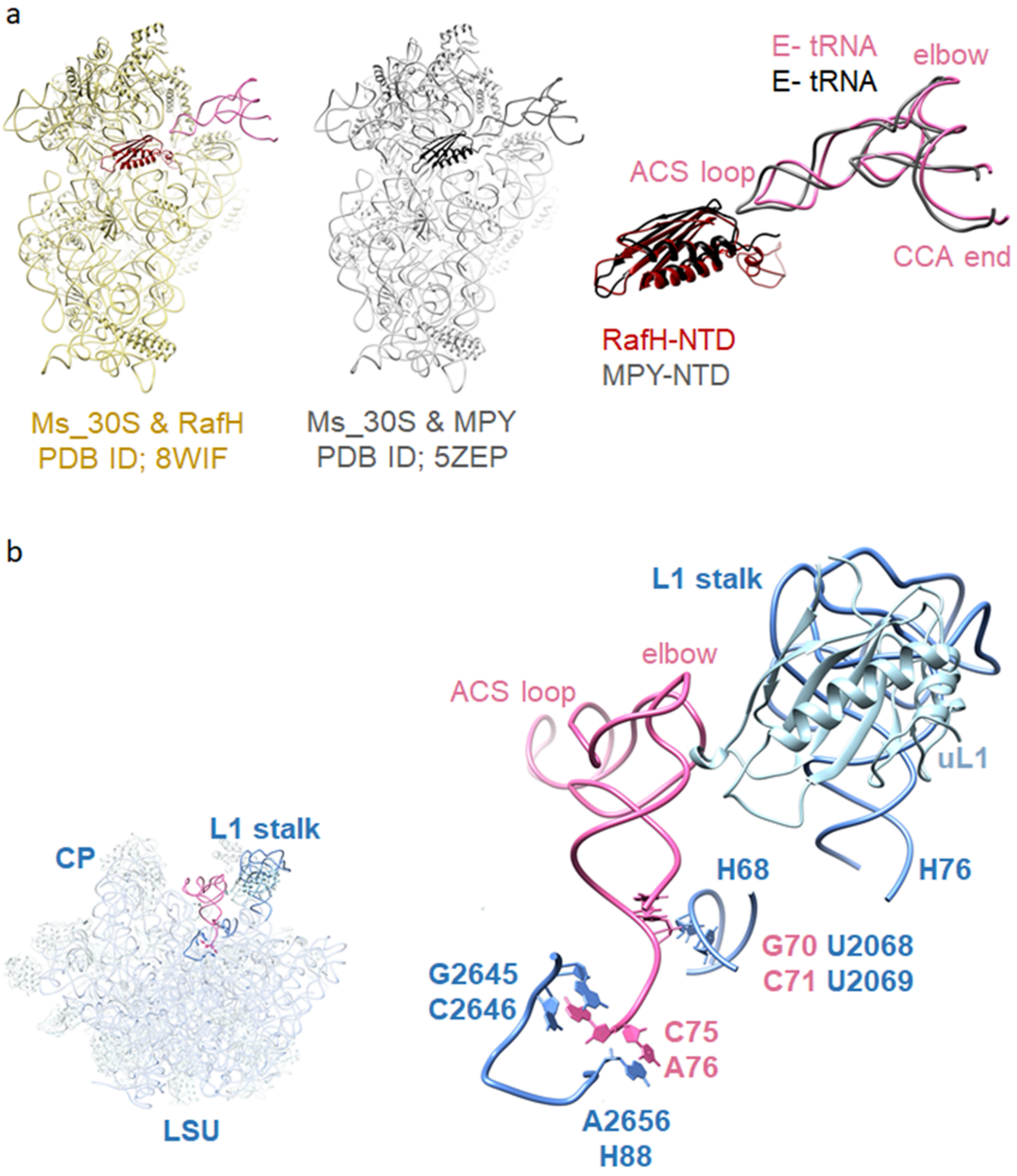

Supplementary Figure 11. E- site tRNA binding analysis. (a) The structures of SSU (Khaki) bound with RafH (red) and E- site tRNA (hot pink) (PDB ID; 8WIF) in left panel, SSU bound with MPY and E- tRNA (grey) (PDB ID; 5ZEP) in the middle panel, and after superimposing the RafH/MPY and tRNAs in the right panel is shown. For clarity, the SSU is not shown in the right panel. (b) E- site tRNA interactions on LSU is shown. The tRNA CCA end-binding pocket, helix H68, tRNA elbow, H76, uL1 r-protein and L1 stalk are labeled. A thumbnail is shown in left panel where CP (central protuberance) and L1 stalk are labeled.

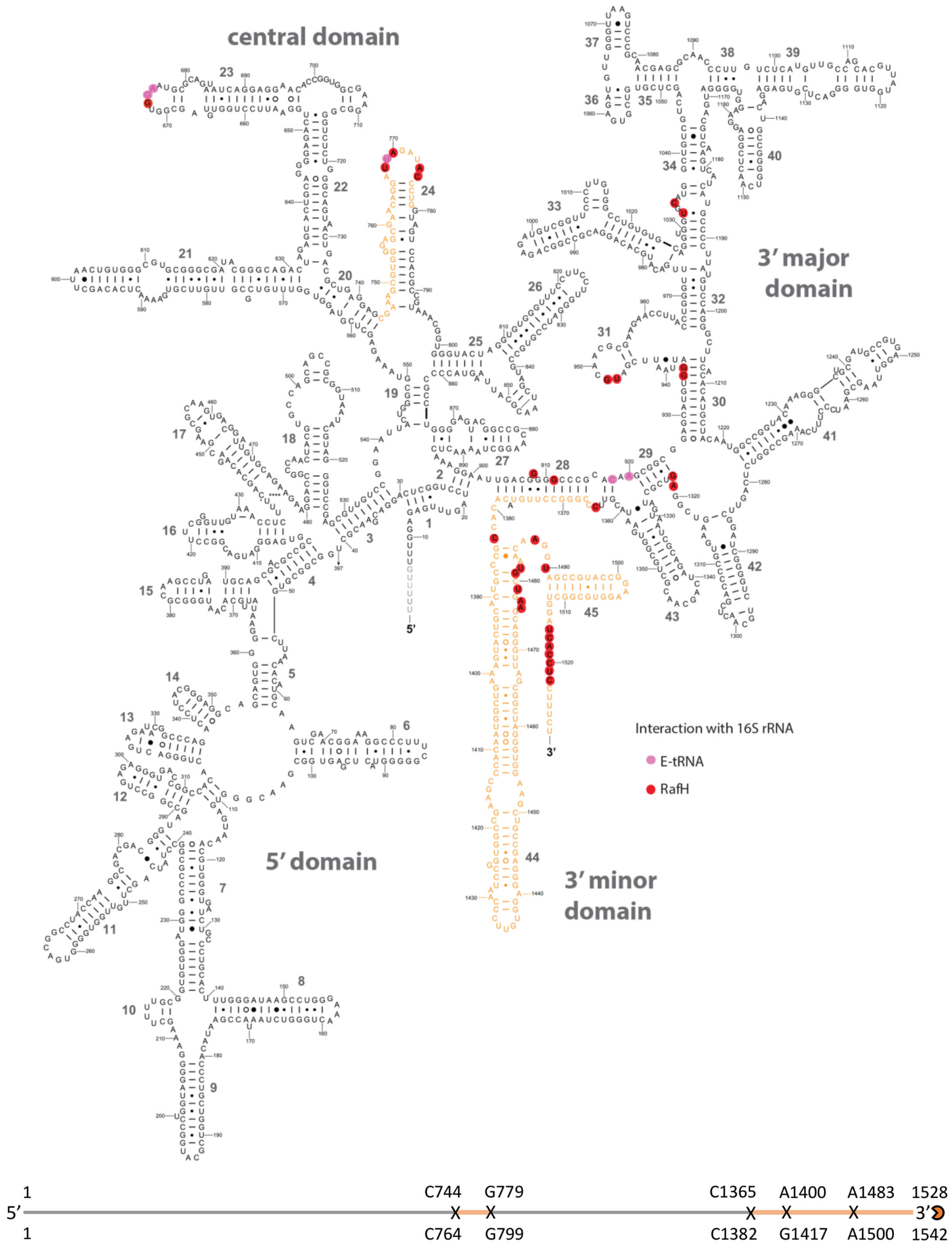

Supplementary Figure 12. 16S rRNA and RNAase degradation sites. (Top panel) the 16S rRNA 2D diagram. The corresponding nucleotide residues prone to RNAase degradation in *M. smegmatis,* as predicted by Prossliner *et al.*, 2022 in *E. coli,* are shown in orange and other nucleotide residues are shown in grey. The RafH and E- tRNA interacting nucleotides are highlighted in red and pink, respectively. The template for 16S rRNA 2D diagram was adopted from Hentschel *et al.*, 2017. (bottom panel) 16S rRNA is shown in a bar diagram. The RNAase endonuclease site in *E. coli* 16S rRNA and the corresponding site in *M. smegmatis* are labeled below and above the bar, respectively. The regions prone to RNAase degradation are shown in orange. 3' to 5' exonuclease RNase PH/RNase R are shown in an orange Pie shape.

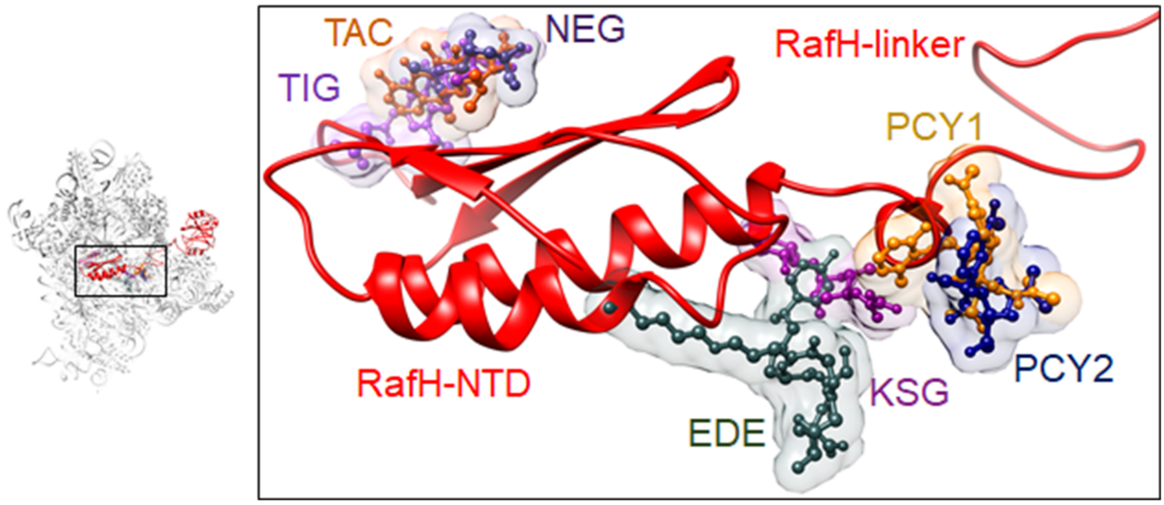

Supplementary Figure 13 RafH NTD and antibiotic binding. The RafH and antibiotic binding in its vicinity on 16S rRNA is shown in the thumbnail on the left side. A magnified view is shown on the right side. The antibiotics were docked onto RafH ribosome 30S. The antibiotics; Tigecycline (TIG) (PDB ID; 4V9B), tetracycline (TAC) (PDB ID; 4V9A), Negamycine (NEG) (PDB ID; 4WF1) Edeine (EDE) (PDB ID; 1I95), Pactamycin1 (PCY1) (PDB ID; 4KHP), Pactamycin2 (PCY2) (PDB ID; 4W2H) and Kasugamycin (KSG) (PDB ID; 4V4H) are shown.

Supplementary Notes 1. List of reagents used in this study.

Reagent	Manufacturer	Catalogue number
HEPES Sodium salt	Sigma aldrich	H3784-500G
TRIS hydrochloride (TRIS-HCl)	Himedia	MB030-1Kg
Tris(hydroxymethyl)aminomethane (Tris, Free Base)	Himedia	MB029-1Kg
Ethylenediaminetetraacetic acid (EDTA) disodium salt dihydrate	Himedia	MB011-500G
Glycine	Himedia	MB013-500G
Acetic acid	Sigma aldrich	A6283
Lauryl sulfate sodium salt(SDS)	Himedia	MB010-500G
Imidazole	Sigma aldrich	I2399-500G
Isopropyl β- d-1-thiogalactopyranoside (IPTG)	Himedia	MB072
Kanamycin sulfate	Sigma aldrich	60615-5G
Magnesium chloride hexahydrate	Sigma aldrich	M2393-500G
Ammonium chloride	Himedia	MB054-500G
Protease Inhibitor cocktail tablets	Roche(Sigma-aldrich)	O5056489001
Phenylmethanesulfonyl –fluoride(PMSF)	Sigma aldrich	P726-25G
D2-Dithiothreitol(DTT)	SRL	17315
Diethyl pyrocarbonate(DEPC)	Himedia	MB076-100ml
Sucrose	Sigma aldrich	S1888-1Kg
Agarose special, low EEO	Himedia	MB002-100G
Sauton's fluid medium base	Himedia	M1276-500G
Luria Broth (LB) medium	Himedia	M1245-500G
Glycerol	Himedia	MB060-1L
Agar powder	Himedia	GRM026-500G
Bis-acrylamide	Himedia	MB005-250G
Acrylamide	Himedia	MB068-1Kg
Ammonium persulfate (APS)	Sigma aldrich	A3678-100G
Ethidium Bromide solution(EtBr)	Sigma aldrich	E-1510-10ml
Coomassie brilliant blue	Sigma aldrich	27816
Bromophenol blue	Himedia	MB123
N,N,N',N'-Tetramethylethylenediamine(TEMED)	Himedia	MB026-100ml
Ni-NTA His-Bind Resin	Merck	7066-5
2-Mercaptoethanol	Sigma-aldrich	M6250-250ml
Tween-20	Himedia	MB067-100mL
Tween-80	Himedia	PCT1513-500mL
Ribonuclease Inhibitor	Sigma	R1158-10KU
DNaseI	Thermo scientific	EN0521
Ethanol	Merck	E1570
Spermidine	Sigma aldrich	S2626

Supplementary Notes 2. Site-directed mutagenesis.

(A) Forward primer W96A- 5’ TATTGCGGAGCAC<u>GCG</u>GAAGCGCGTCG 3’
Reverse primer W96A- 5’ CGACGCGCTTC<u>CGC</u>GTGCTCCGCAATA 3’

```
Sequencing-Msmeg_3935(W96A)    TTANGGNANCNATTTCCCCTCTANAATAATTTTGTTTAACTTTAAGAAGGAGATATACCA    60
Msmeg_3935                     ------------------------------------------------------------    0

Sequencing-Msmeg_3935(W96A)    TGGGCAGCAGCCATCATCATCATCATCACAGCAGCGGCCTGGTGCCGCGCGGCAGCCATA    120
Msmeg_3935                     -----------------------------------------------------------A    1
                                                                                          *

Sequencing-Msmeg_3935(W96A)    TGGATGTGGATGTTAGCACCGATGGCGAACTGCCGGGTGCGGCGGAGTATGCGCGTGAGA    180
Msmeg_3935                     TGGATGTGGATGTTAGCACCGATGGCGAACTGCCGGGTGCGGCGGAGTATGCGCGTGAGA    61
                               ************************************************************

Sequencing-Msmeg_3935(W96A)    AGATTGGTCGTCTGAGCCGTCGTGCGCACCGTCCGGTGCTGCACGCGCGTGTTCGTCTGA    240
Msmeg_3935                     AGATTGGTCGTCTGAGCCGTCGTGCGCACCGTCCGGTGCTGCACGCGCGTGTTCGTCTGA    121
                               ************************************************************

Sequencing-Msmeg_3935(W96A)    CCCGTCACGGTGACCCGGCGGTGGAACGTCCGGTTATCGCGCAGGCGAACCTGGATGTGA    300
Msmeg_3935                     CCCGTCACGGTGACCCGGCGGTGGAACGTCCGGTTATCGCGCAGGCGAACCTGGATGTGA    181
                               ************************************************************

Sequencing-Msmeg_3935(W96A)    ACGGTCGTCAGGTTCGTGCGCAAGTGGAGGGCGTTAACGCGCGTGAAGCGGTGGACCGTC    360
Msmeg_3935                     ACGGTCGTCAGGTTCGTGCGCAAGTGGAGGGCGTTAACGCGCGTGAAGCGGTGGACCGTC    241
                               ************************************************************

Sequencing-Msmeg_3935(W96A)    TGGAGGCGCGTCTGCGTAGCCGTCTGGAGCGTATTGCGGAGCACGCGGAAGCGCGTCGTG    420
Msmeg_3935                     TGGAGGCGCGTCTGCGTAGCCGTCTGGAGCGTATTGCGGAGCACTGGGAAGCGCGTCGTG    301
                               ********************************************  **************

Sequencing-Msmeg_3935(W96A)    GTGGCGTTCCGGCGGAGGCGGGTCGTGAATGGCGTCATGAGAGCGAGCCGGCGCGTCGTC    480
Msmeg_3935                     GTGGCGTTCCGGCGGAGGCGGGTCGTGAATGGCGTCATGAGAGCGAGCCGGCGCGTCGTC    361
                               ************************************************************

Sequencing-Msmeg_3935(W96A)    CGGGCTACTTCCCGCGTCCGCCGGAGGAACGTCGTATCATTCGTCGTAAGAGCTTTAGCA    540
Msmeg_3935                     CGGGCTACTTCCCGCGTCCGCCGGAGGAACGTCGTATCATTCGTCGTAAGAGCTTTAGCA    421
                               ************************************************************

Sequencing-Msmeg_3935(W96A)    TGGTGCCGTGCACCGTTGACGAAGCGGCGCTGGAGATGGAAATGCTGGACTATGATTTCC    600
Msmeg_3935                     TGGTGCCGTGCACCGTTGACGAAGCGGCGCTGGAGATGGAAATGCTGGACTATGATTTCC    481
                               ************************************************************

Sequencing-Msmeg_3935(W96A)    ACCTGTTTACCGAGAAGGGCACCGGTTTTGCGGCGGTGCTGTACAAAGGTGGCCCGACCG    660
Msmeg_3935                     ACCTGTTTACCGAGAAGGGCACCGGTTTTGCGGCGGTGCTGTACAAAGGTGGCCCGACCG    541
                               ************************************************************

Sequencing-Msmeg_3935(W96A)    GTTATCGTCTGGTGCTGGTTATCCCGGTTCCGGCGGATGAGCTGAGCCCGTTTGAAAAAC    720
Msmeg_3935                     GTTATCGTCTGGTGCTGGTTATCCCGGTTCCGGCGGATGAGCTGAGCCCGTTTGAAAAAC    601
                               ************************************************************

Sequencing-Msmeg_3935(W96A)    CGATCACCATTAGCACCCACCCGGCGCCGTGCCTGACCCAACGTGACGCGGTGGAACGTC    780
Msmeg_3935                     CGATCACCATTAGCACCCACCCGGCGCCGTGCCTGACCCAACGTGACGCGGTGGAACGTC    661
                               ************************************************************

Sequencing-Msmeg_3935(W96A)    TGGGTCTGCTGGGCCTGCCGTTCCTGTTTTACATTGATGCGGCGGAGGGCCGTGCGAGCG    840
Msmeg_3935                     TGGGTCTGCTGGGCCTGCCGTTCCTGTTTTACATTGATGCGGCGGAGGGCCGTGCGAGCG    721
                               ************************************************************

Sequencing-Msmeg_3935(W96A)    TTATTTACCGTCGTTATGATGGTCACTATGGTCTGATTACCCCGGCGGATGGTCTCGAGC    900
Msmeg_3935                     TTATTTACCGTCGTTATGATGGTCACTATGGTCTGATTACCCCGGCGGATGGT-------    774
                               *****************************************************

Sequencing-Msmeg_3935(W96A)    ACCACCACCACCACCACTGAGATCCGGCTGCTAACAAAGCCCGAAAGGAAGCTGAGTTGG    960
Msmeg_3935                     ------------------------------------------------------------    774

Sequencing-Msmeg_3935(W96A)    CTGCTGCCACCGCTGAGCAATAACTAGCATAACCCCTTGGGGCCTCTAAACGGGTTCTT    1020
Msmeg_3935                     ------------------------------------------------------------    774

Sequencing-Msmeg_3935(W96A)    GGAGGGGGTTTTTTTGCTGAAAGGAGGAACTATATCCCGGATNGCCGAAATGGGGAC    1080
Msmeg_3935                     ------------------------------------------------------------    774

Sequencing-Msmeg_3935(W96A)    GCGNCCCTGTTAGGCGGGCGCATTTAAAGCGCGGNGGGNNGTGGGTGGNNTACGCGNCAG    1140
Msmeg_3935                     ------------------------------------------------------------    774

Sequencing-Msmeg_3935(W96A)    CGNGACNNCTACANNNNNNAAGCGCCCNTNGCGCCCGCCTCTCNTATNNAGCNTNTTACT    1200
Msmeg_3935                     ------------------------------------------------------------    774

Sequencing-Msmeg_3935(W96A)    TACNCACTNCNT    1212
Msmeg_3935                     ------------    774
```

(B) Forward primer W111A- 5’ GCGGGTCGTGAAGCGCGTCATGAGAGC 3’
Reverse primer W111A- 5’ GCTCTCATGACGCGCTTCACGACCCGC 3’

```
Sequencing-Msmeg_3935(W111A)     TGCGTCATTCCCTCTAGAATAATTTTGTTTAACTTTAAGAAGGAGATATACCATGGGCAG   60
Msmeg_3935                       ------------------------------------------------------------   0

Sequencing-Msmeg_3935(W111A)     CAGCCATCATCATCATCATCACAGCAGCGGCCTGGTGCCGCGCGGCAGCCATATGGATGT   120
Msmeg_3935                       ----------------------------------------------------ATGGATGT   8
                                                                                     ********

Sequencing-Msmeg_3935(W111A)     GGATGTTAGCACCGATGGCGAACTGCCGGGTGCGGCGGAGTATGCGCGTGAGAAGATTGG   180
Msmeg_3935                       GGATGTTAGCACCGATGGCGAACTGCCGGGTGCGGCGGAGTATGCGCGTGAGAAGATTGG   68
                                 ************************************************************

Sequencing-Msmeg_3935(W111A)     TCGTCTGAGCCGTCGTGCGCACCGTCCGGTGCTGCACGCGCGTGTTCGTCTGACCCGTCA   240
Msmeg_3935                       TCGTCTGAGCCGTCGTGCGCACCGTCCGGTGCTGCACGCGCGTGTTCGTCTGACCCGTCA   128
                                 ************************************************************

Sequencing-Msmeg_3935(W111A)     CGGTGACCCGGCGGTGGAACGTCCGGTTATCGCGCAGGCGAACCTGGATGTGAACGGTCG   300
Msmeg_3935                       CGGTGACCCGGCGGTGGAACGTCCGGTTATCGCGCAGGCGAACCTGGATGTGAACGGTCG   188
                                 ************************************************************

Sequencing-Msmeg_3935(W111A)     TCAGGTTCGTGCGCAAGTGGAGGGCGTTAACGCGCGTGAAGCGGTGGACCGTCTGGAGGC   360
Msmeg_3935                       TCAGGTTCGTGCGCAAGTGGAGGGCGTTAACGCGCGTGAAGCGGTGGACCGTCTGGAGGC   248
                                 ************************************************************

Sequencing-Msmeg_3935(W111A)     GCGTCTGCGTAGCCGTCTGGAGCGTATTGCGGAGCACTGGGAAGCGCGTCGTGGTGGCGT   420
Msmeg_3935                       GCGTCTGCGTAGCCGTCTGGAGCGTATTGCGGAGCACTGGGAAGCGCGTCGTGGTGGCGT   308
                                 ************************************************************

Sequencing-Msmeg_3935(W111A)     TCCGGCGGAGGCGGTCGTGAAGCGCGTCATGAGAGCGAGCCGGCGCGTCGTCCGGGCTA   480
Msmeg_3935                       TCCGGCGGAGGCGGTCGTGAATGGCGTCATGAGAGCGAGCCGGCGCGTCGTCCGGGCTA   368
                                 *********************  *************************************

Sequencing-Msmeg_3935(W111A)     CTTCCCGCGTCCGCCGGAGGAACGTCGTATCATTCGTCGTAAGAGCTTTAGCATGGTGCC   540
Msmeg_3935                       CTTCCCGCGTCCGCCGGAGGAACGTCGTATCATTCGTCGTAAGAGCTTTAGCATGGTGCC   428
                                 ************************************************************

Sequencing-Msmeg_3935(W111A)     GTGCACCGTTGACGAAGCGGCGCTGGAGATGGAAATGCTGGACTATGATTTCCACCTGTT   600
Msmeg_3935                       GTGCACCGTTGACGAAGCGGCGCTGGAGATGGAAATGCTGGACTATGATTTCCACCTGTT   488
                                 ************************************************************

Sequencing-Msmeg_3935(W111A)     TACCGAGAAGGGCACCGGTTTTGCGGCGGTGCTGTACAAAGGTGGCCCGACCGGTTATCG   660
Msmeg_3935                       TACCGAGAAGGGCACCGGTTTTGCGGCGGTGCTGTACAAAGGTGGCCCGACCGGTTATCG   548
                                 ************************************************************

Sequencing-Msmeg_3935(W111A)     TCTGGTGCTGGTTATCCCGGTTCCGGCGGATGAGCTGAGCCCGTTTGAAAAACCGATCAC   720
Msmeg_3935                       TCTGGTGCTGGTTATCCCGGTTCCGGCGGATGAGCTGAGCCCGTTTGAAAAACCGATCAC   608
                                 ************************************************************

Sequencing-Msmeg_3935(W111A)     CATTAGCACCCACCCGGCGCCGTGCCTGACCCAACGTGACGCGGTGGAACGTCTGGGTCT   780
Msmeg_3935                       CATTAGCACCCACCCGGCGCCGTGCCTGACCCAACGTGACGCGGTGGAACGTCTGGGTCT   668
                                 ************************************************************

Sequencing-Msmeg_3935(W111A)     GCTGGGCCTGCCGTTCCTGTTTTACATTGATGCGGCGGAGGGCCGTGCGAGCGTTATTTA   840
Msmeg_3935                       GCTGGGCCTGCCGTTCCTGTTTTACATTGATGCGGCGGAGGGCCGTGCGAGCGTTATTTA   728
                                 ************************************************************

Sequencing-Msmeg_3935(W111A)     CCGTCGTTATGATGGTCACTATGGTCTGATTACCCCGGCGGATGGTCTCGAGCACCACCA   900
Msmeg_3935                       CCGTCGTTATGATGGTCACTATGGTCTGATTACCCCGGCGGATGGT--------------   774
                                 **********************************************

Sequencing-Msmeg_3935(W111A)     CCACCACCACTGAGATCCGGCTGCTAACAAAGCCCGAAAGGAAGCTGAGTTTGGCTGCT   960
Msmeg_3935                       ------------------------------------------------------------   774

Sequencing-Msmeg_3935(W111A)     GCCACCGCTGAGCAATAACTAGCATAACCCCTTGGGGCCTCTAAACGGGTCTTGAGGGGT   1020
Msmeg_3935                       ------------------------------------------------------------   774

Sequencing-Msmeg_3935(W111A)     TTTTGCTGAAAGGAGGACTATATCTGATGCGAATGGGACGCCGCCCTGTAGCGCGCAATA   1080
Msmeg_3935                       ------------------------------------------------------------   774

Sequencing-Msmeg_3935(W111A)     GCGCGCGATGTGTGTAACGGCAAGCGTTGAACGGCTACACTTGCCAAGGCGCCCCTTAAG   1140
Msmeg_3935                       ------------------------------------------------------------   774
```
