# Supplementary material for: Cryo- EM structure of the mycobacterial 70S ribosome in complex with ribosome hibernation promotion factor RafH
Source: Nat Commun. 2024 Jan 20;15:638. doi: 10.1038/s41467-024-44879-y (PMC10799931; doi:10.1038/s41467-024-44879-y)
Supplement: Supplementary file 3 — Description of Additional Supplementary Files [file 41467_2024_44879_MOESM3_ESM.pdf]

Description of Additional Supplementary Files

File name: Supplementary Movie 1

Description: An animation for the Figure 2d, created by rotating 360° along Y-axis, where cryo-EM density in mesh and model in stick style corresponds to RafH linker region residues, 111-124 (maroon), and a-SD region of 16S rRNA nucleotides, 1518-1522 (khaki), are shown.

File name: Supplementary Movie 2

Description: An animation for the Figure 3 bottom left panel created by rotating 360° along Y-axis, where cryo- EM density in mesh and model in stick style corresponds to RafH helix α1 R75 (maroon), with Bridge B2a;16S rRNA nucleotides, 1477-1478 (khaki) and 23S rRNA nucleotide A2137 (sky blue), are shown.

File name: Supplementary Movie 3

Description: An animation for the Figure 3 bottom right panel created by rotating 360° along Y-axis, where cryo- EM density in mesh and model in stick style corresponds to RafH helix α1 (maroon) and helix 23s rRNA h44 (khaki), are shown.

File name: Supplementary Movie 4

Description: An animation for the Figure 3 middle right panel created by rotating 360° along Y-axis, where cryo- EM density in mesh and model in stick style corresponds to RafH helix α3 W96 (maroon) and helix 23s rRNA G673 (khaki), are shown.

File name: Supplementary Movie 5

Description: An animation for the Figure 3 top right panel created by rotating 360° along Y-axis, where cryo- EM density in mesh and model in stick style corresponds to RafH helix α2 (maroon) and helix 23s rRNA C1382 and C1383 (khaki), are shown.

File name: Supplementary Movie 6

Description: An animation for the Figure 3 top left panel created by rotating 360° along Y-axis, where cryo- EM density in mesh and model in stick style corresponds to RafH amino acid residues from strands β2, β3, and β4 (maroon) and 23s rRNA h31, U947 and G948 (khaki), are shown.
